# Role of Bacteriophages in the Implementation of a Sustainable Dairy Chain

**DOI:** 10.3389/fmicb.2019.00012

**Published:** 2019-01-22

**Authors:** Diana Gutiérrez, Lucía Fernández, Ana Rodríguez, Pilar García

**Affiliations:** Instituto de Productos Lácteos de Asturias (IPLA-CSIC), Villaviciosa, Spain

**Keywords:** bacteriophage, phage lytic proteins, phage therapy, antibiotic resistance, dairy industry, sustainability

## Abstract

The growing human population is currently facing an unprecedented challenge regarding global food sustainability. Thus, it is of paramount to maintain food production and quality while avoiding a negative impact on climate change and the environment at large. Along the food chain, several practices could compromise future food safety and human health. One example is the widespread use of antibiotics and disinfectants in dairy production, which has contributed to the current antibiotic resistance crisis. Moreover, the uncontrolled release of antimicrobials to the environment poses a significant threat to natural ecosystems. For these reasons, research has recently focused on exploiting natural antimicrobials with the goal of achieving a safer and more sustainable dairy production chain. In this context, bacteriophages, viruses that infect bacteria, may become good allies to prevent and treat diseases in cattle, or be used as disinfectants in dairy facilities and as preservatives in dairy products. This review provides an overview of the current research regarding the use of phages as a global approach to reduce economic losses and food waste, while increasing food safety and reducing the environmental impact of food production. Our current understanding of progress, solutions, and future challenges in dairy production, processing, safety, waste processing, and quality assurance is also discussed.

## Introduction

The current world population is approximately 7.6 billion people and is expected to reach 9.7 billion in 2050 and 11.2 billion in 2100 (UNDESA, 2015). This population growth will proportionally increase global food demands. Such a scenario raises important questions regarding food availability and safety, a phenomenon that is also joined to concerns about water supply, climate change and global sustainability. In fact, sustainability, a complex concept comprising nutrition, safety and sociocultural aspects in addition to availability, accessibility, utilization and stability of food provision, should be considered an integral part of future long-term plans aimed at ensuring food quality and safety (Berry et al., 2015). Indeed, a growing demand for food will likely result in a substantial intensification of production per unit of land and, consequently, put a higher stress on land, water and other natural resources. This would have devastating consequences for natural ecosystems and potentially accelerate climate change.

**FIGURE 1:**
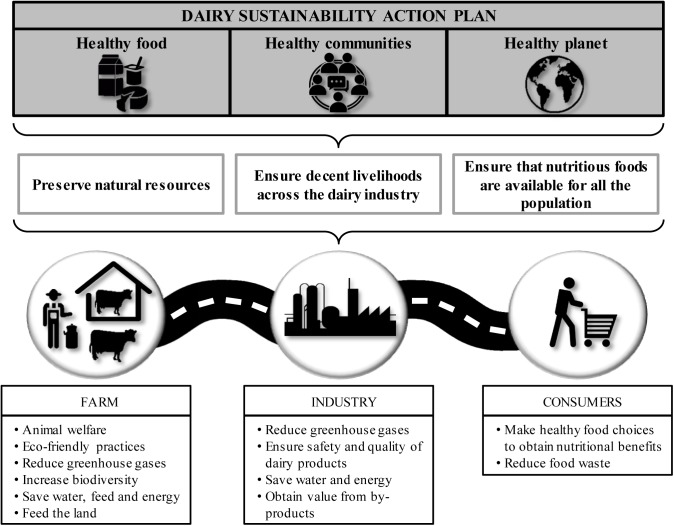
Schematic representation of the dairy sustainability action plan.

In order to attain sustainability, it is necessary to implement a global approach focused on planning, monitoring and evaluating viability along the food chain. This involves an active collaboration between all stakeholders, from farmers, by using eco-friendly practices, to consumers, who should be willing to reduce food waste (Figure 1). Regarding farming practices, a recent analysis about the sustainability of current livestock production systems has shown that the main differences between conventional and organic farms are related to productivity. On the one hand, conventional systems require less labor and land use per product, and exhibit higher production per animal and per time unit, higher reproduction rates, and better udder health for cows. In contrast, organic systems have a lower impact on biodiversity, are less likely to promote antibiotic resistance acquisition in bacteria and increase the beneficial fatty acid levels in cow milk (van Wagenberg et al., 2017). The reduced use of antibiotics in organic farming compared to conventional systems has a clear impact on human health as well as a significant effect on the environment. Indeed, it has been estimated that approximately 75% of the antibiotics used in livestock production are not absorbed by the animals and end up excreted in waste (Chee-Sanford et al., 2009). Moreover, antibiotic resistance selection might occur in gastrointestinal bacteria and result in the accumulation of resistance determinants that will also be excreted by the animals. As a result, land fertilization with manure becomes a source of both antibiotics and genetic resistance determinants (Chee-Sanford et al., 2009). After release to the environment, some antibiotics can persist in soil for a long time where they may be absorbed by plants, frequently with negative consequences. In fact, some antibiotics may negatively affect photosynthesis and oxidative stress responses in plants due to their toxic effect on both chloroplasts and mitochondria (Liu et al., 2011; Moullan et al., 2015).

Antimicrobials are also a problem in the food-processing industries, which constitute an important source of wastewater containing chemical residues of disinfectants and cleaning products. Disinfectants may be toxic for the environment and contribute to the spread of antibiotic-resistance determinants. In fact, there are several examples of cross-resistance between antibiotics and disinfectants (Bore et al., 2007; Hernández et al., 2011). Therefore, the use of natural antimicrobials would diminish the volumes of chemicals that are leaked to the sewage.

Another important goal of a sustainability agenda would be the improvement of food preservation strategies to minimize consumer waste, although in developing countries, the main causes of food spoilage and waste take place at the processing and packaging stages instead of at the consumer stage. In general, fruit and vegetables represent the highest amount of waste, followed by meat, fish, seafood, and milk (Parfitt et al., 2010). Additionally, an important challenge is to avoid the waste of milk containing antibiotic residues above the legal limits (Commission Regulation (EU) No 37/2010 of 22 December 2009). Currently, this milk is used to feed calves but this practice increases the fecal shedding of antimicrobial-resistant bacteria, so that a range of possible options are being considered in order to reduce the concentration of antibiotics in milk (EFSA, 2017).

Here, we will focus on how the dairy chain will need to adapt in order to ensure future supply in the context of a sustainable economy. For example, a higher demand for dairy products will be linked to an increase in resources necessary for raising cattle, including land and water for the production of crops intended for feed production. Additionally, the use of antimicrobials for infection treatment in cattle and for surface disinfection in the dairy industry may have a negative impact on the environment and contribute to the spread of antimicrobial resistance in bacteria. This review will ponder various options for refining the long-term sustainability of milk production with a specific highlight on the replacement of conventional antibiotics and disinfectants by eco-friendly antimicrobials, such as bacteriophages and phage-derived proteins. Moreover, phage-based antimicrobials can be also used for preservation with the aim of reducing waste associated with milk and dairy products.

## Availability of Dairy Products

Milk and dairy products are part of the traditional Mediterranean diet, which is generally regarded as a sustainable and balanced diet thanks to its nutritional quality, health benefits, and low environmental impact (Dernini and Berry, 2015). Indeed, milk and its derived products constitute an important source of proteins, energy and micronutrients (vitamins, calcium, phosphorus, magnesium, zinc, etc.) (Gaucheron, 2011). For this reason, dairy products represent a significant percentage of the total food intake in Western societies while it is growing in developing countries, where the increasing numbers of people’s incomes is changing their diet and consuming a higher amount of dairy products (FAO, 2013). Consequently, the low availability of these products in some parts of the world will be an important challenge for the near future. Overall, the FAO predictions for 2050 highlight the need to increase worldwide agricultural production by 70% compared with 2005 data. Therefore, the sustainability of livestock raising might be seriously affected by competition for natural resources like land and water (Thornton, 2010). In this context, the optimization of animal farming practices, for example by improvement of animal welfare, is essential. Additionally, thinking of a better use of resources, waste of milk and dairy products should be clearly reduced. At the consumer level, the estimated milk waste in industrialized regions represents as much as 40–65% of all food waste and is even higher in developing countries, especially during handling and storage (Burlingame and Dernini, 2010). Therefore, to enlarge the shelf life of dairy products by using new preservation technologies would be a strategy to reduce food waste.

## Risks of Dairy Products Associated With the Proliferation of Pathogenic Bacteria

Generally speaking, milk is widely available in several formats it is inexpensive and safe for most humans, excluding, for example, those with lactose malabsorption and/or intolerance (Ugidos-Rodríguez et al., 2018). Nevertheless, milk and dairy products can become a serious threat to human health when they are contaminated with pathogenic bacteria. This means that ensuring the quality and safety of milk and dairy products along the production chain should be a key point in this type of industries. It is worth noting that the implementation of HACCP practices with the intent of reducing potential microbiological, chemical and physical hazards has had a great impact on the safety of dairy products (van Schothorst and Kleiss, 1994).

Several factors are involved in the microbiological quality of milk: udder health, udder hygiene, and hygienic milk collection (including proper sanitation of equipment and bulk tanks as well as appropriate cooling temperatures). If pathogenic bacteria contaminate milk, the product will pose a food safety threat to consumers (Bender et al., 1999).

In 2016, milk and dairy products constituted the vehicle for 8.6% of all foodborne outbreaks, with *Salmonella* being the most frequently reported causative agent in outbreaks associated with these products (37.7%), followed by *Campylobacter* (22.2%) (EFSA and ECDC, 2017). Moreover, in the US, the number of outbreaks caused by consumption of non-pasteurized milk has increased over the last years^1^. Thus, from 30 outbreaks reported in the period 2007–2009, the number increased up to 51 in 2010–2012, being *Campylobacter* spp. (77%) the main causative agent (Mungai et al., 2015). Products made with raw milk, such as soft cheese, are also the source of outbreaks (Johler et al., 2015). Other important pathogens associated with raw milk consumption are *Listeria monocytogenes, Yersinia enterocolitica* and verocytotoxin-producing *Escherichia coli* (VTEC), and also in endemic areas *Brucella* spp., *Mycobacterium bovis* and the tick-borne encephalitis virus (TBEV) (Verraes et al., 2015). Fecal contamination is an important source of bulk milk contamination for these bacteria since healthy animals can be asymptomatic carriers.

Pathogenic bacteria like *Staphylococcus aureus* and *Streptococcus agalactiae* can also cause subclinical mastitis in herds, which is the first source of milk contamination before processing (Ruegg, 2017). Moreover, these bacteria can spread to the farmers and the bulk tank. If refrigeration conditions are not properly maintained, *S. aureus* can proliferate and synthetize enterotoxins, which will remain in the milk and its derived products even after heat treatment, thanks to their heat-resistant nature, and finally cause food poisoning (Argudín et al., 2010).

During the last years, the dairy industry has made special efforts to improve animal production and reduce the risks associated with pathogenic bacteria. The implementation of quality standards for ‘clean milk’ have progressed and concern about mastitis and its impact on society has expanded to include the effect of mastitis in management programs on farm sustainability and consumer perception. Sanitation practices and the use of antibiotics are tools used to reduce the spread of these bacteria that can evolve from opportunistic pathogens to clinical cases (Ruegg, 2017). Nevertheless, the increase in the last years of bacteria resistant to antibiotics has led to the need for new alternatives to eradicate pathogenic bacteria.

Moreover, pathogenic bacteria such as *L. monocytogenes* and *S. aureus* can be present in the dairy industry during food processing and storage (Latorre et al., 2010; Gutiérrez et al., 2012). Once in the industrial setting, milk is processed using pasteurization or ultra-high-temperature (UHT) treatment. Nevertheless, the presence of bacteria in industrial settings is mainly linked to poor handling practices, changes in temperature and/or cross-contamination. Additionally, bacteria can persist in the batch/processing thanks due to their ability to form biofilms, complex communities where bacteria are attached to a surface and surrounded by a protective extracellular polymeric material. Their extreme resistance to cleaning and disinfecting processes is related to their unique organization, which implies a differential bacterial growth and gene expression inside the biofilm. Biofilm formation of *L. monocytogenes* can be promoted by low pH, salt concentration and temperatures present in dairy industries during cheese manufacturing (Adriao et al., 2008). Also, milk proteins can increase the adhesion of *E. coli*, *S. aureus*, and *L. monocytogenes* to steel food-contact surfaces (Barnes et al., 1999). The impact of biofilms on health, and their economic consequences, has promoted the development of different approaches to control and/or remove biofilms, such as bacteriophages, bacteriocins, quorum-sensing inhibitors, essential oils, high hydrostatic pressure (HHP), non-thermal plasma, enzymatic disruption, steel coatings and photocatalysis (Gutiérrez et al., 2016b; Galié et al., 2018).

## Bacteriophages and Phage Lytic Proteins

**FIGURE 2:**
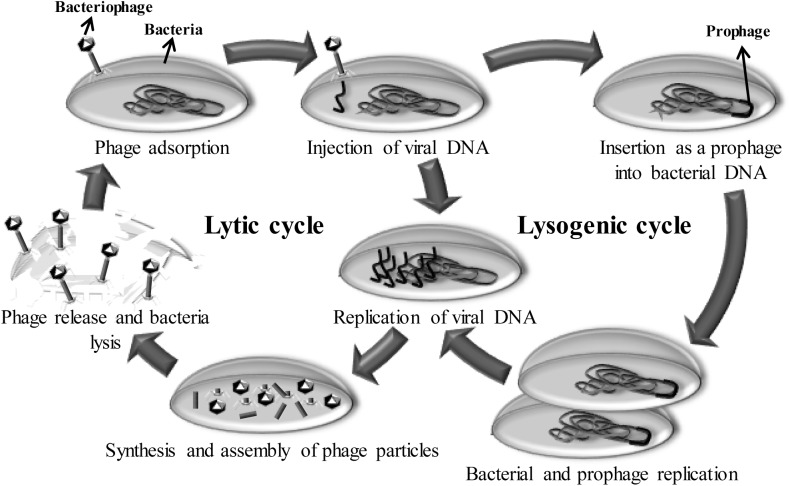
Representation of the bacteriophage life cycles. Bacteriophage life cycles (lysogenic and lytic) start with the adsorption of the phage to the bacterium and continue with the insertion of the viral genetic material into the cytoplasm. At this point, temperate phages (lysogenic cycle) introduce their genetic material (as a prophage) into the bacterial chromosome. Descendant bacterial cells also carry the prophage until the lytic cycle is triggered by an external signal. Upon induction, the lytic cycle continues with the replication of phage genetic material, followed by the synthesis and assembly of viral components. Finally, the bacterial cell is lysed and new phage particles are released.

Bacteriophages (or phages) are viruses that specifically infect bacteria. As obligate parasites, they need a bacterial host to multiply (Figure 2). After infection, phages may follow the lysogenic cycle, where the phage genome will integrate into the host genome (prophage), or the lytic cycle, disrupting the host metabolism and, ultimately, causing the death of the bacterial cell to release new phage particles. Consequently, the life cycle of some phages inherently implies an antimicrobial activity that was discovered at the beginning of the 20th century. At that time, phages were used to treat infectious diseases (phage therapy), although the discovery of antibiotics reduced their implementation in human therapy (Kutter and Sulakvelidze, 2005). Currently, the search for new antimicrobials to combat multi-drug resistant bacteria has led to the reconsideration of phages as tools to fight against pathogenic bacteria. In terms of antimicrobial activity, bacteriophages possess a number of useful properties. For example, they are highly specific for their target bacteria, effective against multi-resistant pathogens and harmless for humans and animals. Indeed, specificity is a very valuable property, as it would prevent interference with the normal microbiota of animals and inhibition of starter cultures in dairy production. Moreover, the auto-replicate ability is a clear advantage comparing with other antimicrobials (O’Flaherty et al., 2009).

**FIGURE 3:**
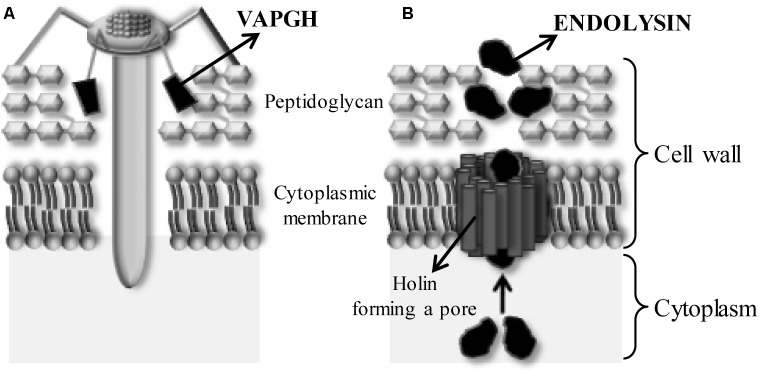
Role of phage lytic proteins when a phage infects a Gram-positive bacterium. **(A)** Action of the virion-associated peptidoglycan hydrolases (VAPGHs) from the outside (‘Lysis from without’) favoring the insertion of phage genetic material by formation of a hole in the cell wall. **(B)** Action of the endolysins from the inside of the bacteria (‘Lysis from within’). Endolysin and holin are produced at the end of the phage life cycle to facilitate release of the new phage particles. Holins form a pore into the bacterial membrane allowing the endolysin to reach the peptidoglycan.

In addition, phages encode some proteins with antimicrobial activity (Figure 3). These enzymes, the so-called peptidoglycan cell wall hydrolases, can be divided into two groups. The first group is composed by the virion-associated peptidoglycan hydrolases (VAPGHs), which are responsible for the initial phage infection step, facilitating the viral DNA entry into the cytoplasm (Figure 3A) and are essential for maintaining the stability of the viral particle (Fischetti, 2005). The other group includes endolysins, proteins that mediate bacterial lysis at the end of the lytic infection cycle (Figure 3B). Both kinds of proteins degrade bacterial peptidoglycan when added externally to the cell, causing the lysis of bacterial cells. These enzymes are named enzybiotics (Fischetti, 2010). Besides these two groups, there exist other phage-structural proteins that can carry catalytic domains with antimicrobial activity, such as the tape measure protein (TMP) (Rodríguez-Rubio et al., 2012b). The antimicrobial activity of these proteins might be used in several areas such as food safety, pathogen detection and diagnosis, surface disinfection, vaccine development and nanotechnology. Similarly to bacteriophages, these proteins are specific against a bacterial genus or even species and their repetitive use does not generate bacterial resistance (Rodríguez-Rubio et al., 2016).

## Applications of Bacteriophages Along the Dairy Chain

**FIGURE 4:**
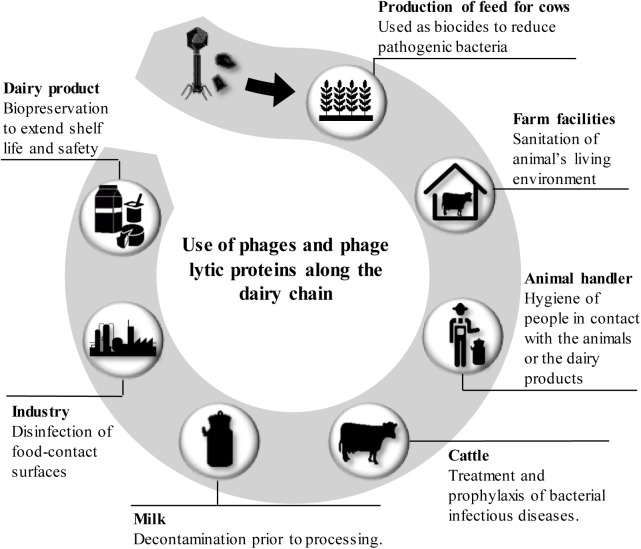
Application of bacteriophages and phage lytic proteins along the dairy chain from ‘farm to fork.’

The specific characteristics of phages and phage lytic proteins make them a feasible alternative to improve the sustainability of dairy products. At this stage, we should mention that, traditionally, bacteriophages have had a negative impact on the manufacture of dairy products as they can infect lactic acid bacteria composing starter cultures with catastrophic consequences for dairy fermentations (Pujato et al., 2019). However, phages infecting pathogenic or spoilage bacteria can be used as new antimicrobials along the dairy chain to prevent problems associated with bacterial contamination (García et al., 2012; Sillankorva et al., 2012; Sulakvelidze, 2013) (Figure 4). In this section, we will gather some of the most important examples of phage applications in the dairy sector that have been examined to date.

### Improvement of Crop Yields

One of the major factors that can jeopardize milk production is the need for meeting the demand for animal feed products. Due to the fact that an important percentage of the total intake of cows is based on grains (barley, corn, oats, and wheat), plant diseases affecting these crops could compromise their availability (Strange and Scott, 2005). Several bacterial phytopathogens have been highlighted due to their relevance in terms of economic losses, such as *Pseudomonas syringae, Ralstonia solanacearum*, *Agrobacterium tumefaciens*, *Xanthomonas, Erwinia amylovora*, and *Xylella fastidiosa* (Mansfield et al., 2012). In order to fight against these pathogenic bacteria, bacteriophages have an eco-friendly status and, in contrast to conventional plant protection products, they are highly specific against their target bacteria. Therefore, they would not be expected to have a negative impact on bacterial biodiversity.

Nowadays, research focused on the use of phages to fight plant pathogens is on the rise (Buttimer et al., 2017) and some products are already available on the market. For instance, Agriphage, a product marketed by Omnilytics, has been designed to fight against *Xanthomonas campestris* pv. *vesicatoria* and *P. syringae* pv. *tomato*, two pathogens that mainly infect tomatoes and peppers. Other examples are Erwiphage, marketed by Enviroinvest against *E. amylovora* for application in apple trees, and Biolyse, which was developed by APS Biocontrol to combat *Enterobacteria* contamination of potato tubers.

Unfortunately, the use of phages is not an option for the inhibition of fungi development (e.g., *Zymoseptoria tritici*, *Triticum aestivum L.*) that negatively affects grain yield and quality. In these cases, several biocontrol approaches have been proposed, such as the use of bacterial endophytes (Huang and Pang, 2017) and other bacteria such as *Bacillus velezensis* (Kang et al., 2018).

### Treatment and Prophylaxis in Cattle

The most prevalent infectious diseases in lactating cows are clinical and subclinical mastitis, metritis, retained placenta, lameness and respiratory diseases. All these illnesses pose an important economic burden to farmers due to reduced milk production, culling increase, and veterinary costs. For example, the estimated cost of each case of clinical mastitis ranges from $95.31 to $211.03 (Cha et al., 2011). Antibiotics have consistently helped to prevent and treat infections in farm animals, but their use has environmental and human health concerns. In this context, the impact of new policies intended to limit antibiotic use in cattle, while being necessary to pave the way toward a “green” economy, can also bear negative consequences. Indeed, Lhermie et al. (2018) have calculated that banning or limiting antibiotic use would significantly raise the cost of dairy production. This cost would vary depending on the disease prevalence but also on cow replacement, cow slaughter and milk prices. In order to alleviate the economic impact of restricting conventional antibiotics, it is necessary to develop suitable alternatives. In this context, bacteriophages and phage lytic proteins seem like a good alternative and/or complement to antibiotic therapy.

Given the high prevalence of mastitis in dairy cows, several studies have assessed the feasibility of using bacteriophages and phage lytic proteins for both prevention and treatment of this disease. So far, most of the work has focused on mastitis caused by *S. aureus* due to the elevated persistence and antibiotic resistance of this pathogen. Initial studies using an intramammary infusion of phage K in mastitic cows were not very encouraging as they showed a curation rate of only 16.7%, with an increase in the milk somatic cell count and with degradation of the infused phage within the gland (Gill et al., 2006). More recently, preliminary results obtained by Fan et al. (2016) have shown that endolysin Trx-SA1 (20 mg once per day) could effectively control mild clinical mastitis caused by *S. aureus* in therapeutic trials carried out on cow udders. Although phages have not shown successful results in cows yet, good results in mice models have been reported, which might indicate the need for more studies. Thus, a work by Breyne et al. (2017) using a mastitic lactating mice model showed a significant improvement on mastitis pathology after treatment with a cocktail consisting of two staphylophages. Phage lytic proteins have also been studied for the treatment of *S. aureus* mammary infections in mice (Schmelcher et al., 2012).

Regarding mastitis caused by *Streptococcus uberis*, valuable research has been recently published by Scholte et al. (2018) showing that concentrations of up to 50 μg/mL of endolysin PlyC did not result in cytotoxicity or oxidative responses on bovine blood polymorphonuclear leukocytes (PMN). Also, another study showed that endolysins from streptococcal phages λSA2 and B30 were effective for the treatment of *Streptococcus*-induced mastitis in mice (Schmelcher et al., 2015).

Mastitis can also be caused by coliforms such as *E. coli*. A candidate to control bovine mastitis is the T4-like virus vB_EcoM-UFV13, which was active against a pathogenic *E. coli* strain in a mastitis mice model (da Silva Duarte et al., 2018).

Another disease that can cause major losses in dairy animal husbandry is calf diarrhea because of its high mortality and morbidity. This disease is often caused by enterotoxigenic *E. coli* (ETEC) strains, many of which display high antibiotic resistance and, consequently, are difficult to treat. In an attempt to overcome this issue, there have been several studies regarding the effectiveness of phages to eliminate this pathogen in calves suffering from experimentally induced *E. coli* diarrhea. For instance, Smith et al. (1987) found that calves could be cured by a single dose of 10^5^ PFU and prevention could be reached by spraying the rooms with a phage suspension containing doses as low as 10^2^ PFU. The results obtained so far indicate that rectal and/or oral treatments of cattle with a phage cocktail can effectively reduce shedding of *E. coli* O157:H7 (Sheng et al., 2006; Rozema et al., 2009). Choosing the right time of application is also a key factor for the success of phage therapy, since the infection of cattle often occurs in calves that are less than 12 weeks old. Therefore, treatment should be applied early in the cattle production cycle. Interestingly, Waddell et al. (2002) found that experimentally infected calves (weaned 7–8-week-old calves) treated orally with a mixture of phages suspended in calf milk replacer resulted in a very high effectiveness and stopped shedding of pathogenic bacteria after 8 days of treatment. Possibly, this was due to the calcium carbonate present in the replacer which can buffer stomach acid and protect viability of the phage particles. Similarly, Stanford et al. (2010) explored polymer encapsulation of four phages in gelatine capsules for oral administration to steers. This strategy did not reduce the numbers of *E. coli* O157:H7 cells released from the animals, but the duration of shedding was shortened to 14 days compared with control steers. Moreover, bacteriophages can be used to avoid transmission to humans of the zoonotic bacterium *E. coli* O157:H7. However, this requires that phage treatment be carried out shortly before slaughter to reduce the risk of carcass contamination.

### Disinfectants for Facilities

Biofilm formation in industrial settings and farms is one of the major sources of contamination with pathogenic or spoilage bacteria. Indeed, most contamination in the dairy industry is due to the persistence of bacterial biofilms following inadequate cleaning of equipment (Latorre et al., 2010). From these biofilms, bacteria can then be easily transmitted to dairy products and ultimately reach consumers. Moreover, biofilms exhibit a high resistance to disinfectants and can often withstand harsh cleaning procedures. Widespread use of disinfectants also has negative consequences due to their environmental toxicity and potential cross-resistance with antibiotics (Gnanadhas et al., 2013). For instance, quaternary ammonium compounds (QACs) have been used for a long time as disinfectants in numerous industrial, medical and domestic applications. As a result, they are common pollutants in waste waters and, although they are biodegradable under some conditions, they are toxic to a lot of aquatic organisms (Zhang et al., 2015). In this regard, multiple studies have explored the use of bacteriophages and phage-derived proteins to improve cleaning and disinfection procedures and, consequently, reduce the risk of bacterial contamination in a more environmentally friendly manner. Bacteriophages have the advantage that they can be easily inactivated before release to environment. Generally, all these studies aim to find new systems for the control of pathogenic bacteria that complement the standard hygiene measures and, consequently, would allow a reduction in the use of chemical disinfectants. However, due to the high specificity of phages and the presence of many different bacterial species, as well as other microorganisms, it is likely that phages can never totally replace the use of disinfectants, but help to improve their effectiveness (Endersen et al., 2014; Gutiérrez et al., 2016b).

There are numerous examples of phages aimed at disrupting biofilms formed by *S. aureus* on food surfaces. Thus, phage K, phage ISP, Romulus, and Remus have all been demonstrated to exhibit the ability to prevent or remove biofilms formed by this pathogen (Vandersteegen et al., 2011, 2013; Kelly et al., 2012; Alves et al., 2014). Interestingly, the antifouling properties of some phages such as phiIPLA-RODI and phiIPLA-C1C against dual-species biofilms (Gutiérrez et al., 2015b; González et al., 2017) varied depending on the accompanying species and the infection conditions. Likewise, research by Fernández et al. (2017) has also shown the importance of using the adequate dose of phages. Indeed, the presence of a non-lethal dose of phage phiIPLA-RODI led to enhanced biofilm formation in *S. aureus* susceptible strains by accumulation of extracellular DNA. The same study showed that low-level phage exposure resulted in transcriptional changes, including upregulation of the stringent response regulon, which could slow down the advance of the bacteriophage within the biofilm.

Phages are also useful for the elimination of other pathogenic bacteria with relevance in the dairy industry such as *L. monocytogenes* (Soni and Nannapaneni, 2010; Nannapaneni and Soni, 2015) and *E. coli* O157:H7 (Sadekuzzaman et al., 2017). The recent availability of several commercial phage-based disinfectants provides evidence of the potential of phages as an attractive complement to classical disinfectants. One recent example is the polyvalent anti-staphylococcal commercial product Stafal^®^, which showed good efficacy for cleaning biofilms formed by methicillin-resistant *S. aureus* in catheters (Dvorackova et al., 2018). Likewise, the commercial product PhageGuard Listex (previously, Listex P100), based on phage P100, has demonstrated anti-listerial activity against biofilms formed by different strains on stainless steel wafers (Iacumin et al., 2016).

The utilization of bacteriophages in combination with other antimicrobials is also an interesting option. Indeed, several studies have reported the synergistic effects between phages and antibiotics for biofilm removal (Kumaran et al., 2018). However, there is still little information about the interactions between phages and biocides. Furthermore, this information is interesting because the use of phages in industrial settings can involve their exposure to disinfectants. Therefore, the outcome of the phage treatment might depend on the susceptibility of the phage to the biocides as well as the existence of synergistic or antagonistic interactions between phages and these chemical agents. For instance, Agun et al. (2018) determined that benzalkonium chloride would be the most adequate disinfectant to apply in combination with the staphylococcal phage phiIPLA-RODI, as this phage can survive at the concentrations of the biocide generally used to inhibit bacterial growth and no antagonism was observed between both antimicrobials.

In addition to bacteriophages, multiple studies have evaluated the use of phage lytic proteins for biofilm removal. For example, there are many endolysins, VAPGH and chimeric lytic proteins with proven ability to disrupt or inhibit the formation of staphylococcal biofilms. These proteins include LysH5 (Gutiérrez et al., 2014), CHAP-SH3b (Fernández et al., 2017), the endolysin from phage phi11 (Sass and Bierbaum, 2007), SAL-2 (an endolysin from bacteriophage SAP-2) (Son et al., 2010), the modified endolysin CHAPk (Fenton et al., 2013), and ClyH (Yang et al., 2014), to name only a few. An interesting observation is that phage lytic proteins such as LysH5 or CHAP-SH3b, did not induce biofilm formation when present at subinhibitory concentrations, a problem frequently observed with some antibiotics and disinfectants (Gutiérrez et al., 2014; Fernández et al., 2017). In fact, they often inhibit biofilm formation, a phenomenon that might be due to the downregulation of genes encoding bacterial autolysins upon exposure to phage lytic proteins (Fernández et al., 2017). Another interesting property of some endolysins is their activity against persister cells and stationary cells as has been shown for LysH5 (Gutiérrez et al., 2014) and the endolysin from phage LM12 (Melo et al., 2018), respectively. Finally, it is worth mentioning that some studies indicate that phage lytic proteins can display better antibiofilm activity than antibiotics (Yang et al., 2014). However, biofilms formed by Gram-negative bacteria are not so easily disrupted. The work published by Oliveira et al. (2014) is an important step forward in the use of endolysins against *Salmonella* biofilms, as they have characterized a high stable endolysin with activity against a wide panel of Gram-negative bacteria in combination with the outer membrane permeabilizers EDTA, citric and malic acid.

Besides directly killing of biofilm cells, it is also convenient to remove the biofilm extracellular material, which is frequently composed of polysaccharides and helps to keep bacteria together and bound to the surface. Some studies have characterized phage-encoded proteins with enzymatic activity against extracellular polysaccharide. For instance, the EPS depolymerase Dpo7, derived from *S. epidermidis* bacteriophage phiIPLA7, showed a maximum removal (>90%) of attached biomass in polysaccharide-producing strains. Moreover, pre-treatment of polystyrene surfaces with Dpo7 inhibited biofilm development so that, in most tested strains, values of biomass were 53–85% lower than those obtained for control cultures grown on untreated surfaces (Gutiérrez et al., 2015a). These results support the potential of this protein to prevent and disperse staphylococcal biofilms, thereby making bacterial cells more accessible to the action of other antimicrobials. Another example is the phage-associated depolymerase Dpo42, encoded by phage vB_EcoM_ECOO78, which displayed antibiofilm activity by degrading the capsular polysaccharides that surround *E. coli* cells (Guo et al., 2017).

Due to the relevance of biofilms in industrial settings, it is essential to develop fast and reliable techniques that allow studying biofilm formation and elimination. Recently, Gutiérrez et al. (2016a) demonstrated that the xCELLigence Real Time Cell Analyzer (RTCA) allows distinguishing biofilm producers from non-producers by monitoring changes in impedance values. Moreover, this technology was later applied for screening and comparing the antibiofilm ability of three phage lytic proteins (LysH5, CHAP-SH3b, and HydH5-SH3b) and an exopolysaccharide depolymerase (Dpo7) against *S. aureus* biofilms (Gutiérrez et al., 2017). This method allowed calculation of the minimum biofilm eradicating concentration that removes 50% of the biofilm (MBEC_50_). This parameter allows comparing the disaggregating activity of different phage antibiofilm proteins (Gutiérrez et al., 2017). With the approach of monitoring biofilm destruction in mind, Tkhilaishvili et al. (2018) used isothermal microcalorimetry, a method that can measure growth-related heat production of microorganisms in real-time. Thus, exposure of *E. coli* biofilms to different titers of T3 bacteriophage (10^2^ to 10^7^ PFU/ml) led to a strong inhibition of heat production, indicating biofilm disruption. This result indicates the feasibility of this strategy for screening the antibiofilm potential of phages.

### Biopreservatives in Dairy Products

As mentioned earlier, waste reduction is an essential point to improve sustainability along the food chain. In the past, most initiatives were focused on reducing food losses during manufacturing, but the current trend is to reduce the food waste generated by consumers, as it regularly reaches 20% of purchased food (Defra and National Statistics, 2017). Within the dairy context, the majority of waste by weight is milk. For example, approximately 360,000 tons of milk are discarded every year in the United Kingdom (WRAP, 2009). In this context, food preservation can help to improve shelf life and, consequently, promote waste reduction. In this regard, the use of both bacteriophages and phage lytic proteins as biopreservatives (Coffey et al., 2010; Mahony et al., 2011) responds to the growing consumer demand for healthy, nutritious, and durable food products, which in turn requires the development of innovative non-thermal food processing methods.

With this in mind, an important body of work has focused on preventing the proliferation of *S. aureus* in milk and dairy products. This bacterium can often contaminate raw milk during the milking process since it can be present in animals suffering from subclinical mastitis or forming biofilms adhered to milking machines. García et al. (2009b) selected two *S. aureus* phages (phiH5 and phiA72) that infected multiple isolates from dairy samples for a preliminary evaluation as biopreservatives in milk. These phages inhibited *S. aureus* growth in UHT and pasteurized whole-fat milk, but were less active in semi-skimmed raw milk and in whole raw milk (García et al., 2009a). Nevertheless, the virulent derivatives (phiIPLA88 and phiIPLA35) obtained from these temperate phages showed higher efficacy. Indeed, a mixture of both virulent phages resulted in complete elimination of the pathogen in UHT whole milk (García et al., 2007). To assess the potential efficacy of a phage-based preservation strategy in pasteurized milk, Obeso et al. (2010) developed a logistic regression model that predicted the net survival/death balance of *S. aureus* cells after 8 h of storage as a function of the initial phage titer, initial bacterial contamination, and temperature. This model indicated that a minimum phage concentration of about 2 × 10^8^ PFU/ml is required for inactivating *S. aureus* in milk stored at different temperatures irrespective of the bacterial contamination level.

In addition to milk, virulent phages phiIPLA88 and phiIPLA35 were also able to rapidly decrease viable counts of *S. aureus* during curd manufacture García et al. (2007). Very importantly, the high specificity of these phages allowed the reduction of pathogenic bacteria in fermented products, such as acid curd or cheese, without disturbing the growth of starter cultures. Indeed, these two phages were successfully evaluated as biocontrol agents in both fresh and hard-type cheeses manufactured with *S. aureus* contaminated milk (Bueno et al., 2012). These bacteriophages were also explored as part of the hurdle concept, using a combination of several preservation technologies aimed at reducing costs while increasing efficacy. Thus, the combined effect of phiIPLA35 and phiIPLA88 together with HHP displayed a synergistic effect and reduced *S. aureus* contamination below the detection limit in pasteurized whole milk under a simulated cold chain break (Tabla et al., 2012). In contrast, the use of bacteriophages in combination with nisin to improve killing *S. aureus* in milk was only recommended for short (<8 h) treatments. Under these conditions, there was a clear synergistic effect but, beyond this time, the development of nisin-adapted cells seriously compromised bacteriophage activity, as they became partially resistant to both phages (Martínez et al., 2008).

The interesting results obtained with this strategy could be extended to another important pathogen associated with contamination of dairy products, *L. monocytogenes*. In this case, the *Siphoviridae* phages LMP1 and LMP7 were able to successfully inhibit growth of this bacterium in milk even at refrigeration temperatures, at which this pathogen is able to grow (Lee et al., 2017). Furthermore, the use of a mixture of phages in combination with the bacteriocin coagulin C23 to reduce the presence of this pathogen in refrigerated milk during storage was assessed. Two *Listeria* bacteriophages belonging to the *Myoviridae* family (FWLLm1 and FWLLm3) that are effective against *L. monocytogenes*, *L. ivanovii*, and *L. welshimeri* (Bigot et al., 2011; Ganegama Arachchi et al., 2013) were shown to act synergistically with coagulin C23 when they were applied in milk contaminated with *L. monocytogenes* and stored at 4°C for 10 days. A combination of phage FWLLm1 (5 × 10^6^ PFU/ml) and coagulin C23 (584 AU/ml) kept *L. monocytogenes* counts under the detection limits (less than 10 CFU/ml) from day 4 until the end of the experiment (Rodríguez-Rubio et al., 2015). In addition to raw milk, *L. monocytogenes* can also contaminate cheese and cause outbreaks. For this reason, Guenther and Loessner (2011) assessed the use of phages to control this pathogen in soft-ripened cheeses (white mold surface or Camembert-type and washed-rind cheese with a red-smear surface or Limburger-type). These authors applied 1 × 10^9^ pfu/cm^2^ of the virulent phage A511 over the surface of a cheese contaminated with 10^1^–10^2^ cfu/cm^2^ of *L. monocytogenes*. Under these conditions, viable counts dropped by more than 6 log units compared to the control, a result that clearly demonstrates the efficacy of this strategy. However, complete removal of *Listeria* was not possible when a higher initial bacterial inocula was used, even after applying successive rounds of treatment.

The influence of the initial contamination level was also corroborated in other soft cheeses. Thus, Minas Frescal and Coalho cheeses were artificialy contaminated with 10^5^ cfu/g of *L. monocytogenes*, and then treated with 8.3 × 10^7^ PFU/g of phage P100 and stored at 10°C for 7 days. Treatment showed some efficacy, as a reduction of about 2 log units in the bacterial counts was detected immediately after inoculation (30 min). Further reduction was lower (0.8 – 1.0 log unit) indicating that a higher phage concentration is required to completely remove the pathogen (Silva et al., 2014).

Bacteriophages can also be an effective control measure against *Salmonella* Enteritidis. In fact, pasteurized whole milk contaminated with 1 × 10^4^ CFU/mL was treated with a cocktail of phages PA13076 and PC2184 (1 × 10^8^ PFU/mL) and incubated at 4°C or 25°C for 5 h. Surprisingly, the inhibitory effect of this phage cocktail was higher at 4°C than at 25°C, with a reduction in bacterial counts of 1.5–4 log units (Bao et al., 2015). Recently, Huang et al. (2018) also showed the ability of phage LPSE1 to decontaminate milk previously contaminated with *Salmonella* Enteritidis by incubation at 28°C with two different MOIs (1 and 100). *Salmonella* cell counts were reduced by approximately, 1.44 log_10_ CFU/mL and 2.37 log_10_ CFU/mL, respectively. Another interesting work published by Tomat et al. (2013) demonstrated that bacteriophages can successfully inhibit the development of pathogenic *E. coli* during milk fermentation. Thus, phages DT1 and DT6 were tested in milk inoculated with *Streptococcus thermophilus* as starter culture, which decreases the pH value to 4–5 after 8 h. Different *E. coli* strains were completely inactivated by this phage cocktail without compromising the starter culture performance. In this context, it is worth noting that *E. coli* strains from dairy origin were only susceptible to phages isolated from the same environment (Molina et al., 2018). Finally, some studies indicate that there is a difference regarding phage performance between UHT and raw bovine milk. McLean et al. (2013) found that a cocktail of three bacteriophages (EC6, EC9, and EC11) completely inhibited *E. coli* O127:H6 in both UHT and raw milk at 25°C and under refrigeration temperatures (5–9°C). However, it was also observed that some *E. coli* strains, such as the enterohemorrhagic strain *E. coli* O157:H7, showed regrowth in raw milk after initial inhibition by the phage cocktail.

Despite the potential of bacteriophages as biopreservatives in food, it is expected that phage lytic proteins have an easier acceptance by consumers and the authorities. Because of that, the use of phage lytic proteins as antimicrobials in a food context is also under investigation (Schmelcher and Loessner, 2016). In a pioneering work, the antimicrobial activity of the phiIPLA88-encoded endolysin LysH5 was proven to rapidly kill *S. aureus* growing in pasteurized milk. Indeed, the pathogen was not detected after 4 h of incubation at 37°C (Obeso et al., 2008). Moreover, the combination of LysH5 and nisin resulted in a strong synergistic effect in pasteurized milk, thereby reducing the amount of antimicrobials necessary to inhibit pathogen development (García et al., 2010). In a similar approach, endolysin LysSA11, derived from the *S. aureus* phage SA11, was assayed as a bactericide but using methicillin-resistant *S. aureus* (MRSA) as a contaminant. Remarkably, the amount of MRSA bacteria (2 × 10^5^ CFU/mL) in milk was significantly reduced (1.44-log CFU/mL) after 15 min even at refrigeration temperatures, although the reduction was lower than at room temperature (2.02-log CFU/mL). In any case, the viable cells were rapidly reduced to undetectable levels by treatment with 9 μM LysSA11 (Chang et al., 2017a). Furthermore, to facilitate the potential application of LysH5 during the manufacture of fermented dairy products, the endolysin was expressed and secreted in *Lactococcus lactis* using the signal peptide of lactococcin 972 as well as lactococcal constitutive and inducible promoters. *L. lactis* is a GRAS (generally regarded as safe) microorganism used in food fermentations which could be suitable as a starter and/or protective culture in dairy fermentations, as well as for protein secretion in large-scale production processes and downstream purification steps (Rodríguez-Rubio et al., 2012a). In a similar work (Guo et al., 2016), used endolysin Lysdb, encoded by the *Lactobacillus delbrueckii* phage phiLdb, to remove *S. aureus* cells as this endolysin was able to cleave the 6-*O*-acetylated peptidoglycans present in the cell walls of *S. aureus*. Thus, Lysdb was constitutively delivered using *Lactobacillus casei* BL23 constructs, which were used in cheese making from raw milk. During the fermentation process, the viable counts of *S. aureus* were reduced by 10^5^-fold in the cheese inoculated with the *L. casei* strain, and then remained at a low level (10^4^ CFU/g) after 6 weeks of ripening at 10°C. Besides endolysins, the antimicrobial activity of a VAPGH encoded by phage phiIPLA88, named HydH5, was improved by shuffling its domains with lysostaphin to obtain three different fusion proteins, which showed higher staphylolytic activity than the parental enzyme (Rodríguez-Rubio et al., 2012c). Remarkably, all catalytic domains present in these proteins were active, and resistant *S. aureus* could not be identified even after 10 cycles of bacterial exposure to phage lytic proteins either in liquid or plate cultures (Rodríguez-Rubio et al., 2013b). These interesting properties led the authors to assess the lytic activity of these chimeric proteins in commercial milk. Thus, the chimeric protein CHAP-SH3b showed a high staphylolytic protection as it was able to eradicate staphylococcal contamination at room temperature by addition of 1.65 mM (Rodríguez-Rubio et al., 2013a). Exploring synergistic effects between endolysins and essential oils (Chang et al., 2017b), used LysSA97, an endolysin encoded by the staphylococcal bacteriophage SA97, and carvacrol. The synergistic antimicrobial activity showed a higher bacteria reduction (4.5 ± 0.2 log CFU/mL) for the mixture of two antimicrobials than for the individual use of either one. However, this synergistic activity appeared to be influenced by the total lipid content of foods, therefore, bacteria in skim milk were more drastically inactivated than those in whole milk, probably due to the fact that essential oil components were less soluble and bound to fat globules in whole milk.

Synergistic effects between endolysins and other preservation techniques were also exploited for the removal of *L. monocytogenes*. Thus, the combination of endolysin PlyP825 and HHP against this bacterium in milk and mozzarella cheese was examined by Misiou et al. (2018). This study found that the application of PlyP825 prior to HHP processing allowed cell inactivation, and then a reduction in the pressure level with equal antimicrobial efficacy. Remarkably, a pressure level of 200 MPa combined with PlyP825 was sufficient to reduce the number of viable bacteria, whereas in mozzarella cheese higher pressure (400 MPa) was required due to the lower activity of the endolysin. Also, using a synergistic approach (Ibarra-Sánchez et al., 2018), found that endolysin PlyP100 was stable in Hispanic-style fresh cheese for up to 28 days of cold storage. Therefore, the combination with nisin (250 μg nisin + 2.5 U PlyP100 per g of cheese) was able to reduce 10^4^ CFU/g of *L. monocytogenes* to cell counts below the detection limit after 28 days, without development of nisin-resistant cells.

## Future Perspectives

The use of antibiotics should be drastically reduced throughout the dairy chain in order to limit the spread of antimicrobial resistance in pathogenic bacteria. In this context, bacteriophages and phage lytic proteins have a great potential to substitute or complement more traditional antimicrobials. Of course, extensive research needs to be performed before their widespread use is implemented. In particular, it is essential to identify possible short- and long-term risks associated with phage application in the environment (e.g., application in the countryside and farms), including the potential development of bacterial resistance. Nonetheless, our current knowledge, including the extensive experience accumulated through therapeutic use in Eastern Europe, supports the idea that rational use of phages can help to mitigate the antibiotic resistance crisis. Moreover, phage-based antimicrobial strategies fit perfectly within the framework of the goals compiled in the 2030 Agenda for Sustainable Development, in which the EU made a positive and constructive contribution^2^. This Agenda proposes a plan of action that considers the well-being of mankind and planet a like by seeking, among other objectives, the achievement of food security through sustainable agriculture (Goal 2) and protection of ecosystems (Goal 15).

Moreover, the Global Dairy Agenda for Action (GDAA), which was signed by seven organizations representing participants in the dairy sector from farm through processing to the factory gate from all around the world, also has a sustainability plan for this sector. Overall, phages as natural antimicrobials can offer a very target-specific environment-friendly and safe alternative to chemicals and help us achieve the human health and environmental protection objectives that will lead us into a better future.

## Author Contributions

PG conceived and designed the work. PG, LF, DG, and AR wrote the manuscript.

## Conflict of Interest Statement

The authors declare that the research was conducted in the absence of any commercial or financial relationships that could be construed as a potential conflict of interest.

## Footnotes

1 https://www.cdc.gov/foodsafety/rawmilk/rawmilk-outbreaks.html

2 https://ec.europa.eu/europeaid/policies/european-development-policy/2030-agenda-sustainable-development_en
